# Safety and feasibility of adjuvant chemotherapy with S-1 in Japanese breast cancer patients after primary systemic chemotherapy: a feasibility study

**DOI:** 10.1186/s12885-015-1289-7

**Published:** 2015-04-10

**Authors:** Takashi Shigekawa, Akihiko Osaki, Hiroshi Sekine, Nobuaki Sato, Chizuko Kanbayashi, Hiroshi Sano, Hideki Takeuchi, Shigeto Ueda, Noriko Nakamiya, Ikuko Sugitani, Michiko Sugiyama, Hiroko Shimada, Eiko Hirokawa, Takao Takahashi, Toshiaki Saeki

**Affiliations:** 1 Department of Breast Oncology, Saitama Medical University International Medical Center, 1397-1 Yamane, Hidaka-shi, Saitama 350-1298 Japan; 2 Department of Radiation Oncology, Saitama Medical University International Medical Center, Saitama, Japan; 3 Department of Breast Oncology, Niigata Cancer Center Hospital, Niigata, Japan; 4 Department of Surgery, Sasaki Memorial Hospital, Saitama, Japan

**Keywords:** Advanced breast cancer, S-1, Adjuvant chemotherapy, Primary systemic chemotherapy

## Abstract

**Background:**

Advanced breast cancer patients have a higher risk of postoperative recurrence than early-stage breast cancer patients. Recurrence is believed to be caused by the increase in micrometases, which were not eradicated by preoperative or postoperative chemotherapy. Therefore, a new therapeutic strategy that can improve treatment efficacy is mandatory for advanced breast cancer. S-1 was shown to be effective and safe in Japanese metastatic breast cancer patients treated with previous chemotherapy, including anthracyclines. Thus, in this study, we evaluated S-1 as adjuvant chemotherapy in breast cancer patients after standard primary systemic chemotherapy.

**Methods:**

The treatment consisted of 18 courses (a 2-week administration and a 1-week withdrawal; one year) administered at 80–120 mg/body/day. In cases judged to require postoperative radiotherapy, it was concurrently initiated on Day 1 of the study. If the estrogen receptor and/or human epidermal growth factor receptor 2 were positive, endocrine therapy and/or trastuzumab were permitted, concurrently.

**Results:**

Of the 45 patients enrolled between September 2007 and September 2009 from 3 institutions, 43 patients were eligible. Thirty-two of the 43 (74.4%) patients received concurrent radiotherapy. Twenty-two of the 43 (51.2%) patients completed the scheduled courses of chemotherapy. The most common reasons for withdrawal of treatment were subjective symptoms, such as nausea, anorexia, or general fatigue during the first 9 courses of treatment in 9/43 (20.9%) patients, recurrence in 7/43 (16.3%) patients, and adverse events in 5/43 (11.6%) patients. The cumulative percentage of administration for 365 days was 66.4% (95% confidence interval: 50.8–79.1%). Although grade 3 neutropenia (9.3%), leukopenia (4.7%), and diarrhea (4.7%) were observed, they were manageable. No grade 4 adverse effects were observed.

**Conclusions:**

The percentage of Japanese breast cancer patients completing the 18-course treatment and the cumulative percentage of administration for 365 days using S-1 after standard primary systemic chemotherapy were similar with the results of another study of adjuvant chemotherapy for the Japanese gastric cancer patients with no severe adverse effects. A phase III trial investigating the usefulness of adjuvant S-1 is now ongoing in Japan, and it is expected that S-1 will have a significant survival benefit in breast cancer patients.

UMIN000013469.

## Background

Primary systemic chemotherapy (PSC) is recommended as a standard therapy in locally advanced breast cancer and, at present, it is the standard of care for early-stage breast cancer [1]. Advanced breast cancer patients have a higher risk of postoperative recurrence than early-stage breast cancer patients. The recurrence is believed to be caused by the growth of micrometases, which could not be controlled by standard treatment. Therefore, a new therapeutic strategy that can improve the treatment effect is mandatory for advanced breast cancer. S-1 is a dihydropyrimidine dehydrogenase (DPD)-inhibitory fluoropyrimidine (DIF), and it is combined with gimeracil, oteracil, and tegafur in a molar ratio of 1:0.4:1. S-1 was shown to be effective and safe in Japanese metastatic breast cancer patients treated with previous chemotherapy, including anthracyclines [2]. Another oral fluoropyrimidine-based regimen, tegafur/uracil (UFT), was proven to be effective as adjuvant chemotherapy in Japanese breast cancer patients [3]. In addition, adjuvant chemotherapy with S-1 was useful in gastric cancer patients [4]. Thus, S-1 is expected to be a promising drug that may have a survival benefit in the adjuvant setting. We evaluated the safety and feasibility of adjuvant chemotherapy with S-1 for curatively resected advanced breast cancer patients after standard PSC. There have been reports of S-1 with concurrent radiotherapy in several types of cancer patients [5,6]; therefore, concurrent administration was planned and performed in patients judged to require postoperative radiotherapy.

## Methods

### Design of the study

This was a multicenter, non-blinded, open-label, feasibility study. The primary endpoints of this study were the percentage of the eligible patients completing the 18-course treatment and the cumulative percentage of administration for 365 days using S-1. The secondary endpoint was safety. In PSC, the clinical response was evaluated by ultrasonography according to the Response Evaluation Criteria In Solid Tumors (RECIST) version 1.1., and the pathological response was assessed according to the criteria established by the Japanese Breast Cancer Society. In the criteria, pathological complete response (pCR) was defined as necrosis and/or disappearance of all tumor cells, and/or the replacement of cancer cells by granulation and/or fibrosis. If only ductal components remained, the pathological response was described as a pCR. The adverse events were evaluated with Common Terminology Criteria for Adverse Events v3.0 (CTCAE v3.0) and the frequency of the worst grade was reported. In this trial, on the basis of the annual number of patients receiving PSC followed by surgery, we determined feasibility. The sample size was estimated to be approximately 40 patients, without any calculations based on statistical assumptions.

### Patient eligibility criteria

Patient eligibility criteria for this study were as follows: (1) breast cancer with histological confirmation; (2) Stage II or III breast cancer (AJCC Cancer Staging Manual. 7th edition) and previous standard anthracycline and taxane-based PSC, followed by curative surgery; (3) age 20–75 years; (4) Eastern Cooperative Oncology Group performance status 0–1; (5) adequate gastrointestinal function; (6) adequate organ function [leukocytes ≥4000/mm^3^; neutrocytes ≥2,000/mm^3^; platelets ≥100,000/mm^3^; hemoglobin ≥9.0 g/dl; serum total bilirubin ≤1.5 mg/dl; aspartate aminotransferase (AST) and alanine aminotransferase (ALT) <2.5 times the normal limits at each institution; and serum creatinine, ≤1.5 mg/dl].

Patients with serious complications, a history of drug hypersensitivity, brain metastases with any symptoms, active secondary cancer, or pregnant women were excluded. This study was performed in accordance with the ethical principles of the Declaration of Helsinki and was approved by the institutional review board of all participating hospitals (Saitama Medical University International Medical Center, Niigata Cancer Center Hospital, and Sasaki Memorial Hospital). Informed consent was obtained from all patients prior to enrollment in this study by written.

### Treatment schedule

36 patients (83.7%) received PSC with epirubicin 90 mg/m^2^ and cyclophosphamide 600 mg/m^2^ q3w followed by docetaxel 75 mg/m^2^ q3w. 3 patients (7.0%) received PSC with docetaxel 75 mg/m^2^ q3w followed by epirubicin 90 mg/m^2^ and cyclophosphamide 600 mg/m^2^ q3w. In 4 patients (9.3%), PSC with epirubicin 90 mg/m^2^ and cyclophosphamide 600 mg/m^2^ q3w was administered, and because of the toxicity of anthracycline, it was impossible to continue docetaxel for them. Adjuvant chemotherapy consisted of 18 courses (a 2-week administration and a 1-week withdrawal; one year) of S-1 (tegafur, gimeracil, and oteracil potassium; Taiho Pharmaceutical, Tokyo, Japan) administered orally at 80–120 mg/body/day, twice daily after breakfast and dinner, according to the body surface area (BSA). Patients with BSA <1.25 m^2^ received 80 mg/day. Patients with BSA ≥1.25 and <1.5 m^2^ received 100 mg/day. Patients with BSA ≥1.5 m^2^ received 120 mg/day. The treatment was started within 28 days after curative surgery. In cases judged to require postoperative radiotherapy, concurrent administration was performed. Treatment was initiated on Day 1 of the study. A fractional daily dose of 2.0 Gy (5 days/week), up to a total dose of 50.0 Gy, was prescribed, and boost radiation of 10.0 Gy/5 fr was permitted, if needed. In estrogen receptor and/or progesterone receptor (ER and/or PgR)-positive cases, endocrine therapy (premenopausal, tamoxifen; postmenopausal, letrozol) was permitted concurrently. In human epidermal growth factor receptor 2 (HER2)-positive cases, trastuzumab was also permitted concurrently. Treatment was discontinued when the patient had recurrence of disease or adverse reactions unable to be controlled by dose modification or temporary withdrawal of S-1. Treatment was also discontinued at patient request. Adverse events were assessed using the National Cancer Institute Common Toxicity Criteria (version 3.0).

### Dose modification

S-1 was temporally discontinued until recovery when any of the following conditions were encountered: leukocytes, <2000/mm^3^; neutrocytes, <1000/mm^3^; platelets, <75,000/mm^3^; hemoglobin, <8.0 g/dl; serum total bilirubin, ≥3.0 mg/dl; serum AST/ALT, >150 IU/L; serum creatinine, >1.5 mg/dl, or when grade 2 or higher non-hematological toxicity occurred. The permitted period of withdrawal because of adverse reactions was <14 days. If patients had hematological or non-hematological toxicities of ≥ grade 3, their daily dose was reduced from 120 to 100 mg, 100 to 80 mg, or 80 to 50 mg, and the dose reduction was permitted several times until 50 mg before treatment interruption.

### Follow-up

Patients underwent hematological tests and assessments of clinical symptoms at least once during each course of chemotherapy. Recurrence was diagnosed on the basis of imaging studies, mainly chest and abdominal computed tomography and bone scans, which were performed at 6- and 12-month intervals, respectively, during the first 2 years after surgery. After that, regular medical history reviews and physical examinations were performed every 6–12 months, and mammography was performed every 12 months according to the American Society of Clinical Oncology clinical practice guideline [7].

### Statistical analysis

The cumulative percentage of administration for 365 days using S-1 was evaluated using the Kaplan–Meier method, and a 95% confidence interval (CI) was calculated.

## Results

### Patient characteristics

Among the 45 patients enrolled from 3 institutions between September 2007 and September 2009, 2 patients were found to be ineligible after enrollment; 1 patient withdrew consent to enter this trial, and the other patient underwent a non-curative operation. The demographic and clinical characteristics of the 43 eligible patients are summarized in Table 1. Thirty-two of the 43 (74.4%) patients received concurrent radiotherapy. Endocrine therapy was concurrently administered in 26 of the 43 (60.5%) patients with S-1. In 2008, after trastuzumab was available for use in an adjuvant setting by a medical service under health insurance, trastuzumab was concurrently administered with S-1 in 2 of the 43 (4.7%) patients.

**Table 1:** **Demographic and clinical characteristics of the 43 eligible patients**

Characteristics	No. of patients
Eligible patients	43
Gender	
Female	43
Age (years)	
Median	53
Range	32-71
ER status	
Positive	28(65.1%)
Negative	15(34.9%)
PgR status	
Positive	17(39.5%)
Negative	26(60.5%)
HER2 status	
Positive	5(11.6%)
Negative	38(88.4%)
BSA	
<1.25 m^2^	0(0%)
1.25 m^2^ ≤ <1.50 m^2^	19(44.2%)
1.5 m^2^≤	24(55.8%)
Menopausal states	
Premenopausal	22(51.2%)
Postmenopausal	21(48.8)
Histological classification	
Invasive ductal carcinoma	41(95.4%)
Invasive lobular carcinoma	1(2.3%)
Apocrine carcinoma	1(2.3%)
Stage	
IIA	1(2.3%)
IIB	17(39.5%)
IIIA	4(9.3%)
IIIB	6(14.0%)
IIIC	15(34.9%)
Surgery	
Mastectomy	24(55.8%)
Partial resection	19(44.2%)
Concurrent radiotherapy	
Yes	32(74.4%)
No	11(25.6%)
Concurrent drug	
Yes	28(65.1%)
Tamoxifen	16(37.2%)
Letrozol	10(23.3%)
Trastuzumab	1(2.3%)
Tamoxifen + Trastuzumab	1(2.3%)
No	15(34.9%)
PSC regimen	
Anthracycline-based regimen followed by taxane regimen	36(83.7%)
Anthracycline-based regimen	4(9.3%)
Taxane regimen followed by anthracycline-based regimen	3(7.0%)
Clinical response for RPC	
CR	3(7.0%)
PR	26(60.4%)
SD	11(25.6%)
PD	3(7.0%)
Pathological response for RSC	
Non-p CR	38(88.4%)
p CR	5(11.6%)

### Feasibility

**Figure 1:**
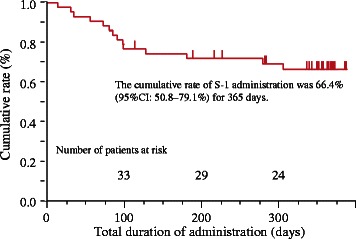
Cumulative percentage of S-1 administration for 365 days. Censoring ticks show the patients who discontinued S-1 because of adverse event, patient refusal, or recurrence.

Compliance of S-1 for each course in the eligible patients and in the patients without recurrence is shown in Table 2. As shown in Table 2, 22 of the 43 (51.2%) eligible patients completed the 18-course treatment. Reasons for discontinuing treatment were patient refusal (9 patients, 20.9%), recurrence of disease (7 patients, 16.3%), and adverse reactions (5 patients, 11.6%). The cumulative percentage of administration for 365 days was 66.4% (95% CI: 50.8–79.1%) (Figure 1). The compliance of the 18-course treatment was 2 of 5 (40%) patients in S-1 alone group, 4 of 12 (33%) patients in S-1 with radiation group, 5 of 6 (83%) patients in S-1 with endocrine therapy group, and 11 of 20 (55%) patients in S-1 with radiation and endocrine therapy group. As shown in Table 2, the 9 patients who refused to continue in the study did so during the first 9 courses. All 9 patients discontinued because of subjective symptoms, such as nausea, anorexia, or general fatigue. Of those 9, 7 (77.8%) patients received concurrent radiotherapy between the first 2 courses, 6 (66.7%) patients received concurrent endocrine therapy, and 5 (55.6%) patients received both concurrent radiotherapy and endocrine therapy. None of the patients received trastuzumab concurrently. The other reasons for discontinuation of treatment were the detection of recurrence in 7 patients and the decision of the investigators to terminate treatment because of adverse events or complications in 5 patients. Of the 5 patients who discontinued because of adverse events or complications, 2 (1 in S-1 with radiation and endocrine therapy group and 1 in S-1 with radiation group) had myelosuppression, 2 (1 in S-1 with radiation and endocrine therapy group and 1 in S-1 with radiation group)had elevated liver function, and 1 (in S-1 with radiation and endocrine therapy group) had diarrhea. The dose of S-1 was decreased in 8 of the 43 (18.6%) patients. Of the 22 patients who completed 18 courses of chemotherapy, the dose was decreased in 6 patients.

**Table 2:** **Compliance of S-1 for each course**

Cycle number	Completion rate in the eligible patients	Reasons for discontinuation of treatment (The number of patients)
1	97.7%(42/43)	Adverse event(1)
2	93.0%(40/43)	Patient refusal(2)
3	90.7%(39/43)	Adverse event(1)
4	86.0%(37/43)	Patient refusal(2)
5	74.4%(32/43)	Patient refusal(3)
		Adverse event(1)
		Recurrence(1)
6	69.8%(30/43)	Patient refusal(1)
		Recurrence(1)
7	69.8%(30/43)	
8	69.8%(30/43)	
9	62.8%(27/43)	Patient refusal(1)
		Recurrence(2)
10	32.8%(27/43)	
11	60.5%(26/43)	Recurrence(1)
12	60.5%(26/43)	
13	55.8%(24/43)	Adverse event(1)
		Recurrence(1)
14	51.2%(22/43)	Adverse event(1)
		Recurrence(1)
15	51.2%(22/43)	
16	51.2%(22/43)	
17	51.2%(22/43)	
18	51.2%(22/43)

### Toxicity

The adverse reactions experienced by the 43 patients are listed in Table 3. Neutropenia, leukopenia, thrombocytopenia, anemia, elevated liver function, anorexia, general fatigue, diarrhea, nausea, stomatitis, and pigmentation changes were comparatively frequent. Although grade 3 neutropenia (4 patients; 9.3%), leukopenia (2 patients; 4.7%), and diarrhea (2 patients; 4.7%) were observed, they were manageable. No grade 4 adverse events were observed. There were no skin reactions of ≥ grade 3 in the surgical wound, although that was a concern in patients treated with concurrent radiotherapy.

**Table 3:** **Adverse reactions (n = 43)**

	Grade					
	1	2	3	4	Total	
	n	n	n	n	n	(%)
Hematological toxicities						
Neutropenia	7	8	1	0	19	(44.2%)
Leukopenia	5	21	2	0	28	(65.1%)
Thrombocytopenia	13	0	0	0	13	(30.2%)
Anemia (Hb)	11	3	0	0	14	(32.6%)
AST	12	0	0	0	12	(27.9%)
ALT	11	0	0	0	11	(25.6%)
Total bilirubin	7	1	0	0	8	(18.6%)
Creatinine	2	0	0	0	2	(4.6%)
Non-hematological toxicities						
Anorexia	24	4	0	0	28	(65.1%)
General Fatigue	29	2	0	0	31	(72.1%)
Diarrhea	13	0	2	0	15	(34.9%)
Nausea	12	4	0	0	16	(37.2%)
Stomatitis	15	1	0	0	16	(37.2%)
Pigmentation changes	17	3	0	0	20	(46.5%)
Rash	3	1	0	0	4	(9.3%)

## Discussion

Meta-analyses have proven that the survival rates of preoperative and postoperative chemotherapy are equal. In recent years, preoperative chemotherapy has become common for operable breast cancer patients [8,9]. In the NSABP B-18, B-27, and Aberdeen trials, patients who achieved a pathological complete response (pCR) had better disease-free survival and overall survival than those who did not [10,11]. In the NSABP B-27 trial, anthracycline-based regimens followed by taxane regimens had higher pCR rates than anthracycline-based regimens [12]. Thus, in this study, we chose an anthracycline-based regimen followed by a taxane regimen as the standard PSC. As a new therapeutic strategy for advanced breast cancer that can improve the treatment effect, we performed this first pre-stage feasibility study of a randomized trial using S-1 in an adjuvant setting.

S-1 is a DIF. Another DIF, UFT, was proven to be effective as adjuvant chemotherapy in Japanese breast cancer patients [3]. S-1 is the drug combined with gimeracil, oteracil, and tegafur in a molar ratio of 1:0.4:1. Tegafur was originally synthesized as a prodrug of 5-fluorouracil (5-FU) and is converted to 5-FU in the liver by cytochrome P450 2A6. 5-FU is promptly metabolized by DPD in the liver; therefore, tegafur was combined with uracil (UFT) or gimeracil (S-1) to inhibit DPD and to increase the concentration of 5-FU. Gimeracil inhibits approximately 200-fold DPD of uracil. Oteracil suppresses the activation of 5-FU, mainly in the gastrointestinal tract, and decreases gastrointestinal toxicity. Thus, S-1 is considered to have high antitumor activity and low gastrointestinal toxicity [13]. Advanced breast cancer can respond to S-1. In a phase II trial, S-1 showed a high efficacy (an overall response rate of 41.7%) with low toxicity [2]. S-1 is expected to be a promising drug, which may have a survival benefit in the adjuvant setting. In Japanese gastric cancer patients, it was reported that adjuvant chemotherapy with S-1 was useful [4]. Thus, it led to the idea of using S-1 as an adjuvant chemotherapy for curatively resected advanced breast cancer patients after standard primary systemic chemotherapy. It was reported that S-1 with concurrent radiotherapy has been used to treat several types of cancer patients [5,6]; thus, concurrent administration was planned and performed in patients judged to require postoperative radiotherapy.

This is the first report examining adjuvant chemotherapy for breast cancer with S-1 after standard PSC. In this study, we chose the schedule of a 2-week administration and a 1-week withdrawal based on a report in pancreatic cancer patients [5]. We felt that a 4-week continuous medication schedule, without a rest, may be too toxic for heavily pretreated breast cancer patients after standard PSC. In this feasibility study, there are the following methodological limitations that because no statistical hypothesis was planned, this feasibility study was exploratory, generating and no demonstrating an hypothesis of safety and feasibility of adjuvant chemotherapy with S-1, and that because of the small sample size and the wide eligibility criteria it was impossible to identify patients at particularly high/low risk of bad/good feasibility.

In our trial, the primary endpoints were the percentage of the eligible patients completing the 18-course treatment and the cumulative percentage of administration for 365 days using S-1, and they were 51.2% and 66.4% (95% CI: 50.8–79.1%), respectively. Compared to the results of another study of adjuvant chemotherapy for the Japanese gastric cancer patients [14], in which the planned courses of S-1 (a 4-week administration and a 2-week withdrawal for 1 year) were administered in 60.7% of the patients, our result was acceptable. According to the results, no grade 4 adverse effects were observed. One drawback in this study was the high incidence of patient refusal of the treatment during the first 9 courses (the first half) because of subjective symptoms, such as nausea, anorexia, or general fatigue. Those symptoms were all grade 1 or grade 2. This problem (in the first half) may have been caused by the influence of the adverse effects of PSC, surgery, and the concurrent radiotherapy in the first 2 courses, or the concurrent endocrine therapy. In this study, although treatment with S-1 was started within 28 days after curative surgery, a delay in the start of drug administration may be necessary to prevent this problem in the early courses. The administration of radiotherapy and S-1 sequencially instead of concurrently may yield a better result. Considering that the concurrent use of S-1 and endocrine therapy was feasible without any refusal and with a few adverse event (only 2 cases) in the 9 cycles of the latter half, the concurrent use of S-1 and endocrine therapy may be acceptable. A randomized phase III trial comparing adjuvant S-1 plus standard hormonal therapy to standard hormonal therapy alone in ER-positive and HER2-negative primary breast cancer with intermediate and/or high risk of recurrence is now ongoing in Japan (POTENT: Postoperative Therapy with Endocrine and TS-1, UMIN000003969). In this study, S-1 is administered using the same schedule as our study (a 2-week administration and a 1-week withdrawal for 1 year) with the concurrent endocrine therapy. It is expected that S-1 will have a significant survival benefit in breast cancer patients.

## Conclusions

In our study, the percentage of the eligible patients completing the 18-course treatment and the cumulative percentage of administration for 365 days using S-1 were similar with the results of another study of adjuvant chemotherapy for the Japanese gastric cancer patients, and there were no severe adverse effects. A phase III trial investigating the usefulness of concurrent S-1 and endocrine therapy in the adjuvant setting is now ongoing in Japan. S-1 is expected to have a significant survival benefit in breast cancer patients.

## Abbreviations

PSC: Primary systemic chemotherapy
ER: Estrogen receptor alpha
PgR: Progesterone receptor
BSA: Body surface area
HER2: Human epidermal growth factor receptor 2
CI: Confidence interval
pCR: Pathological complete response
DPD: Dihydropyrimidine dehydrogenase
DIF: DPD-inhibitory fluoropyrimidine
UFT: Tegafur/uracil
5-FU: 5-Fluorouracil
